# Glow Up Pediatric Minor Trauma Care: Detecting Adrenal Bleeding with Contrast-Enhanced Ultrasound in Emergency Settings

**DOI:** 10.24908/pocusj.v10i02.19131

**Published:** 2025-11-17

**Authors:** Yair Katzir, Yehuda Tzur

**Affiliations:** 1 Pediatric Emergency Department, Hillel Yaffe Medical Center, Hadera, Israel; 2 Pediatric Department, Hillel Yaffe Medical Center, Hadera, Israel; 3 Technion, Israel Institute of Technology, Haifa, Israel

**Keywords:** Trauma, Abdominal point-of-care ultrasound, Contrast enhanced ultrasound, Pediatric emergency department

## Abstract

Minor abdominal blunt trauma is a common pediatric emergency department presentation. Contrast-enhanced computed tomography (CECT) is the current gold standard imaging modality for identifying abdominal parenchymal injuries. Contrast-enhanced ultrasound (CEUS) has been suggested as a radiation-sparing alternative. Here we describe, for the first time, the use of pediatric emergency physician-performed CEUS in the evaluation of minor abdominal trauma in a child. The use of CEUS informed the decision-making process in this case and ultimately led to the diagnosis of an adrenal hemorrhage.

## Initial Case Presentation

A 3.5-year-old previously healthy boy presented with two episodes of non-bilious and non-bloody emesis to the pediatric emergency department six hours following a minor abdominal trauma. The patient had fallen from a standing height onto a seesaw, with the seesaw bar hitting the right side of his abdomen.

Vital signs were all within the normal range (blood pressure 109/65 mmHg, heart rate 108 bpm, blood oxygen saturation 99%). Physical examination was notable for mild tenderness at the right upper quadrant (RUQ) of his abdomen. There was no obvious bruising, abrasions or hematoma at the site of injury. The abdomen was soft and non-distended.

Peripheral intravenous (IV) access was established. A complete blood count displayed a normal hemoglobin of 12.2 g/dL. His coagulation studies were within normal limits for his age.

His comprehensive metabolic panel was notable for a mild elevation of aspartate transaminase of 60 U/L (normal range 9-31 U/L), with no concomitant elevation of alanine transaminase. His urinalysis was negative for blood.

## Ultrasound Findings

**Figure 1.**
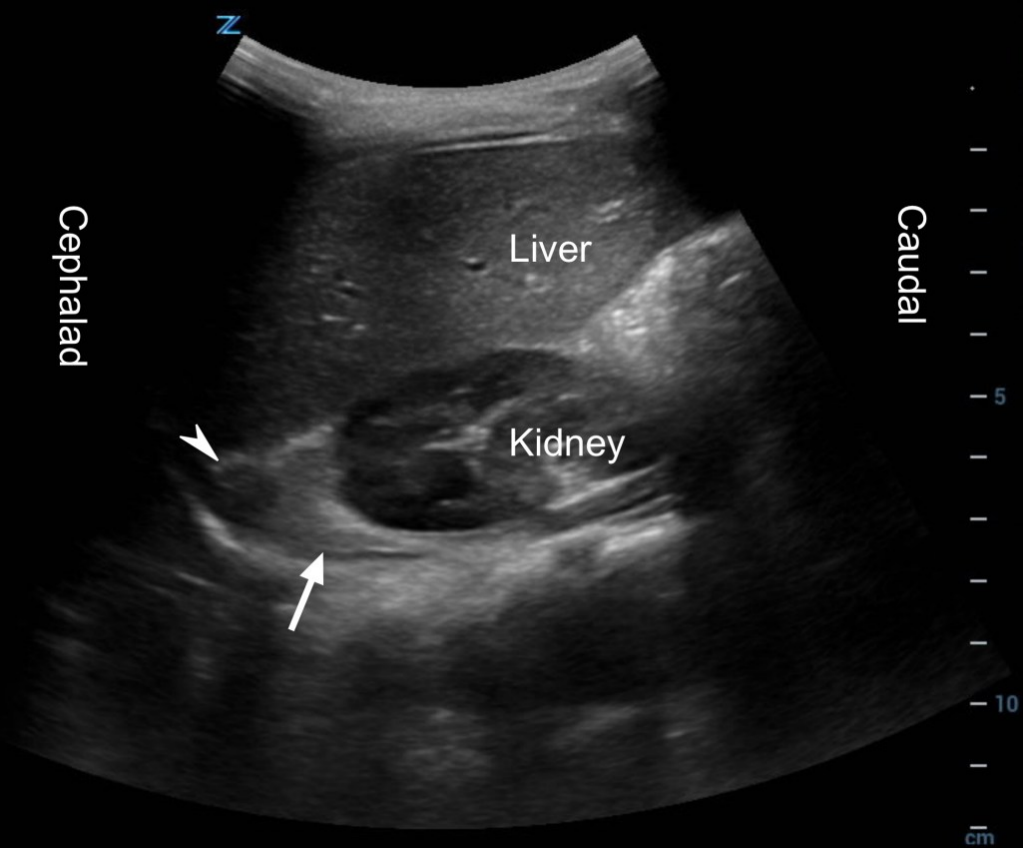
A focused assessment with sonography in trauma (FAST) right upper quadrant (RUQ) standard view. A prominent adrenal gland area (arrow) and an oval finding (arrowhead), later characterized as a blood clot, are seen in the cephalad pole of the kidney.

An initial focused assessment with sonography in trauma (FAST) examination and interval FAST after two hours of observation were both negative for free fluid. While performing the RUQ portion of the initial FAST exam, a hypoechoic ovoid finding (2.2 cm X 1.7 cm) that was roughly isoechoic to the liver was seen in the border of the upper pole of the kidney and the prominent adrenal gland (Figure 1). These findings were not indicative of free fluid, which appears either anechoic or hypoechoic, and were not present on the contralateral side.

**Figure 2.**
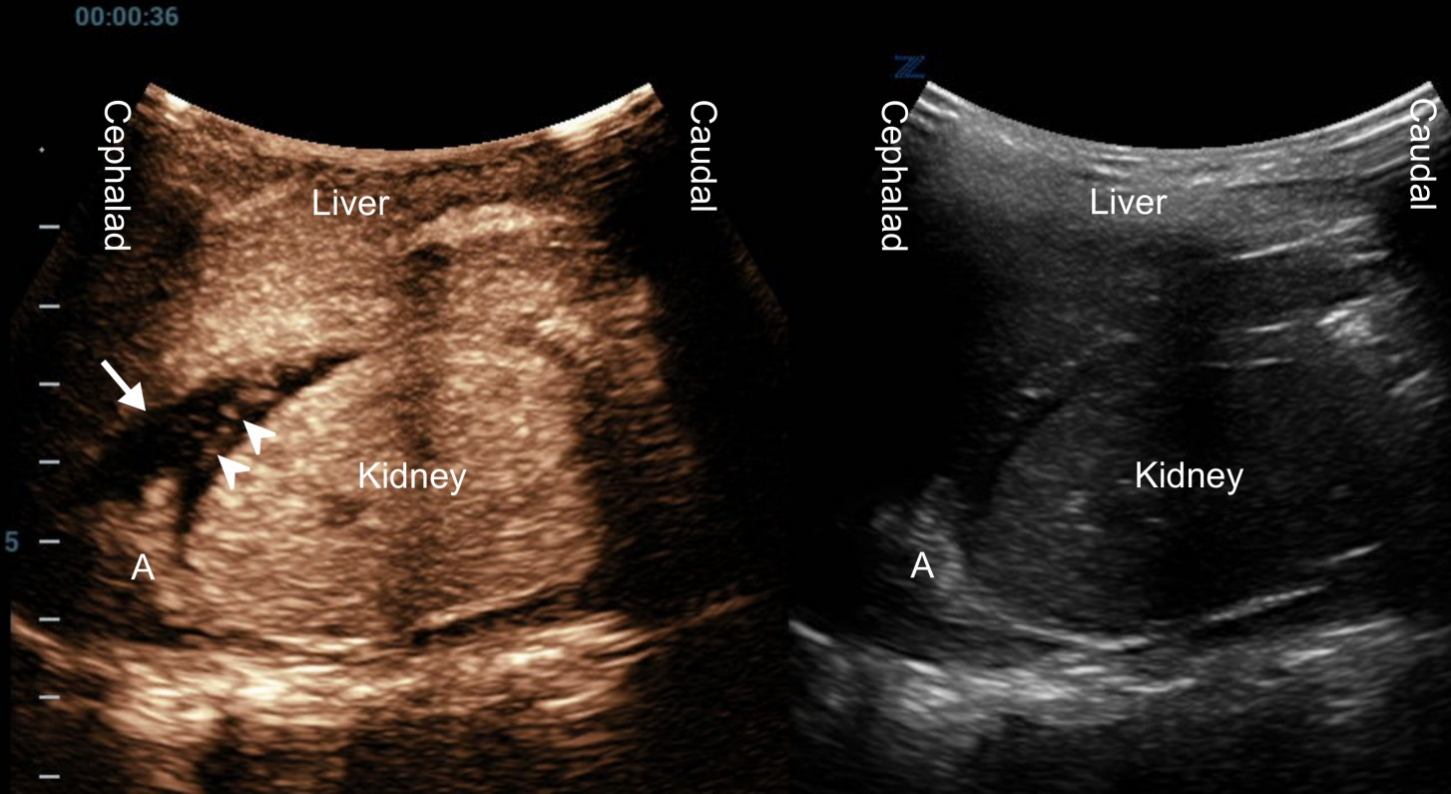
A dual-mode contrast-enhanced ultrasound (CEUS) image of the same (Figure 1) focused assessment with sonography in trauma (FAST) right upper quadrant (RUQ) view (left side - CEUS; right side - grayscale) demonstrating adrenal (A) bleeding. The hematoma appears non-enhancing hypoechoic (arrow), and the extravasation of bubbles (arrowheads) indicates active bleeding.

**Figure 3.**
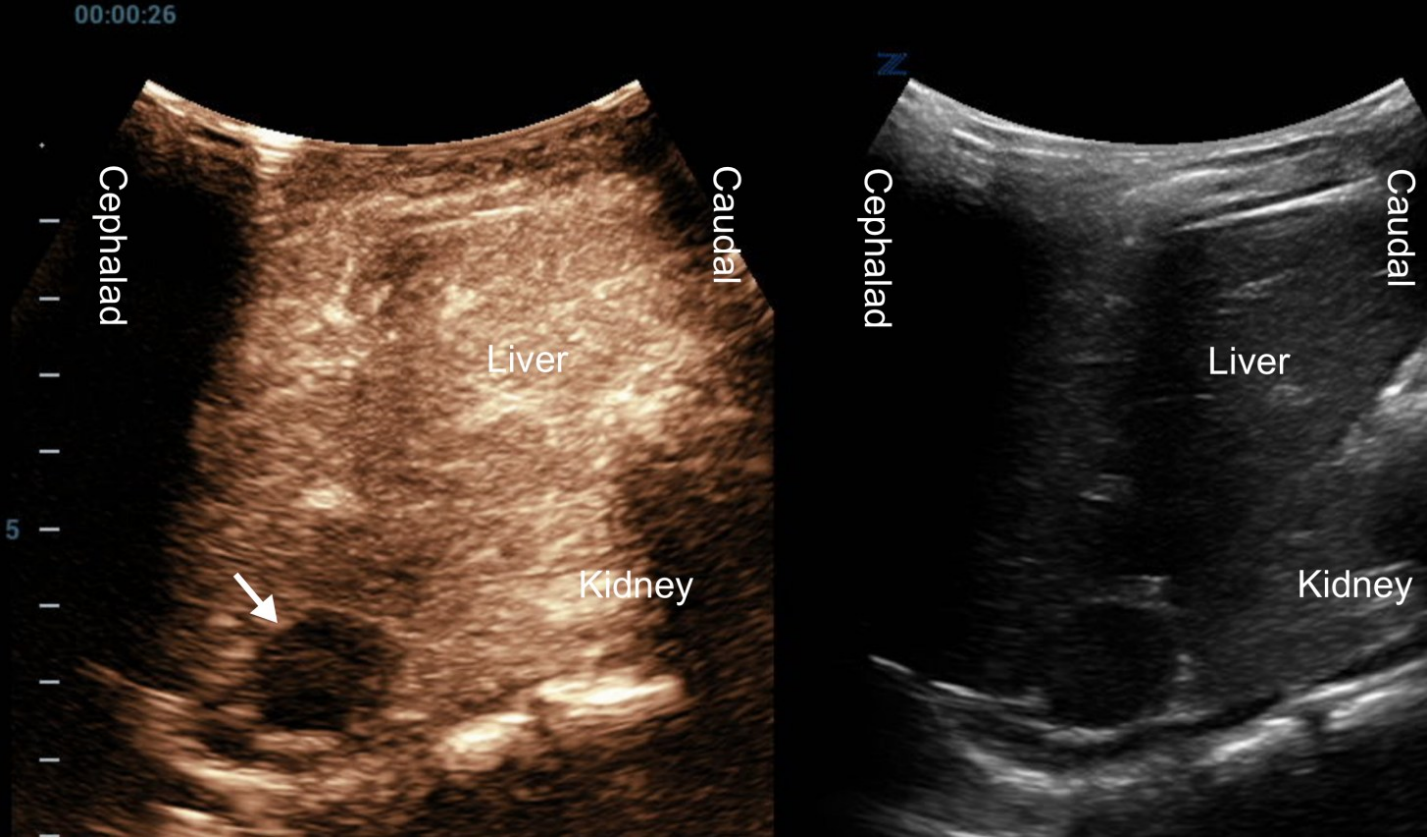
A dual-mode contrast-enhanced ultrasound (CEUS) image (left side - CEUS; right side - grayscale), at the arterial phase, of the oval finding (arrow) at the adrenal cephalad area. The kidney appears in a longitudinal view. This finding shows no marked enhancement, suggesting a blood clot.

A follow-up CEUS was conducted by the first author—a pediatric emergency medicine attending physician specializing in point of care ultrasound (POCUS) with CEUS training at King's College Hospital, London, United Kingdom. The CEUS scan revealed a triangular area lacking enhancement in the pre-renal space, indicative of a large hematoma adjacent to the adrenal gland. Blood was extravasating into the hematoma, as evidenced by a stream of enhanced bubbles from the gland into the evolving hematoma (Figure 2 and Supplementary Material S1). The oval finding seen on non-contrast POCUS was demonstrated on CEUS, with no marked enhancement (Figure 3). It was observed in both the arterial phase (20-40 seconds after injection) and the late phase (parenchymal, >40 seconds after injection). The main differential diagnosis for this finding was a blood clot or other space-occupying lesion. Vascularized lesions exhibit a characteristic enhancement pattern at the arterial phase and wash-out at the parenchymal phase according to their vascularity. In contrast, a non-vascularized lesion exhibits low and constant enhancement throughout the phases [1]. The appearance of the lesion seen here, on both the arterial and late phases, showed low and constant enhancement and was thus more indicative of a clot [2].

## Ultrasound Technique

Modern ultrasonography contrast agents (UCAs), such as SonoVue (marketed as Lumason in the USA), consist of microbubbles with a gas core and a stabilizing shell. A CEUS is performed by injecting UCAs intravenously or intracavity.

CEUS mode is based on the separation between the linear reflection of the ultrasound signal by tissues and the non-linear reflection induced by oscillations of the microbubble UCA. This distinction is a key aspect of CEUS.

Using a low mechanical index (MI) is instrumental in CEUS. MI is related to the peak acoustic pressure of the ultrasound waves. Low MI effectively minimizes microbubble destruction and tissue harmonics, thereby enhancing the contrast-to-tissue ratio and reducing artifacts.

The order of scanning is a derivative of the enhancement and clearance rates of the UCA for the abdominal parenchymal organs. The order of scanning in this case followed trauma CEUS protocols, starting with the kidney and adrenal area on the affected side, then proceeding to the kidney on the contralateral side, the liver, and finally, the spleen [3]. Images were obtained by a Zonare ultrasound machine (Z.One pro, Mindray, formerly ZONARE Medical Systems inc., Mountain View, CA, US) with a curvilinear 1- to 6-MHz transducer. CEUS images were recorded in dual mode (contrast/B-mode) with Zonare CEUS-specific software. An intravenous injection of 0.6 mL of the UCA, SonoVue (Bracco SpA, Milan, Italy), administered via a 22-gauge peripheral line with a 3-valve extension, followed by a rapid 5 mL flush of saline [4].

## Case Progression

Due to the concerns identified on POCUS, the child was sedated and a CECT scan was performed. CECT confirmed the presence of a large hematoma in the right adrenal gland, with no evidence of active bleeding, likely due to the higher flow threshold for detection in CECT compared to CEUS. After a surgical consultation, the child was transferred to a pediatric intensive care unit in a tertiary hospital with interventional radiology services available. A total decrease of 1 g/dL in the patient's hemoglobin level was observed, which stabilized at 11.2 g/dL. The child improved with conservative treatment alone and was discharged later from the pediatric intensive care unit without any invasive intervention. A follow-up ultrasound was scheduled 14 days after discharge. This was conducted by a pediatric radiologist who characterized the lesion as a dissolving blood clot.

## Discussion

Abdominal blunt trauma is a common reason for pediatric emergency department encounters and is a leading cause of morbidity in children [5]. While the FAST examination in adults has a sensitivity of 85-96% for detecting hemoperitoneum, the FAST examination used in the pediatric population may have significantly lower sensitivity (50%) [6,7]. Recent studies indicate better results (sensitivity up to 89%) with high-quality scans and serial FAST exams [8,9]. However, non-contrast ultrasound exhibits low sensitivity for detecting parenchymal injuries, even among experienced operators [10]. Therefore, CECT remains the gold standard for abdominal blunt trauma in children [10]. Given the resource burden and the radioactive harms associated with CECT, particularly among the pediatric population who are more susceptible to the deleterious effects of ionizing radiation, there is increasing interest in the clinical applications of CEUS [11,12]. In recent years, CEUS scans performed by radiologists have been shown to possess accuracy nearly equivalent to that of CECT for parenchymal and visceral injuries [10,13]. FAST examinations combined with CEUS performed by POCUS-experienced emergency physicians have a very high sensitivity for intra-abdominal injuries in stable adult patients presenting with abdominal trauma, but no equivalent studies discussing CEUS performed by pediatric emergency physicians have been published to date [14].

We use CEUS in our pediatric emergency department as a decision-making tool in one of two scenarios. First, when there is low clinical suspicion of parenchymal injury but a computed tomography (CT) scan is indicated. Thus, with a negative CEUS scan, we avoid performing a CT scan. The other scenario is described here—when there is a low indication for CT. However, there is a discrepancy between at least two of the following three elements: the mechanism of injury, the child's clinical presentation, and the child's objective findings (laboratory results, FAST, etc.). In this case, the focal finding on the FAST exam conflicted with the mechanism and clinical presentation, so we turned to CEUS for further evaluation. A positive result prompted a CECT for confirmation, while a negative would have steered us away from a CT scan. The positive identification proved necessary for appropriate management of the child's intra-abdominal injury.

## Conclusions

To our knowledge, this is the first reported case in which a CEUS scan was conducted in the pediatric emergency department by pediatric emergency physicians, leading to a significant impact on clinical decision-making. CEUS may serve as a safer alternative to CECT as a screening imaging tool in abdominal blunt trauma.

## References

[1] Piskunowicz M, Stefanowicz J, Batko T, Hwang M, Świętoń D, Szarmach A, Back S, Kosiak W. Contrast-enhanced ultrasound of adrenal hemorrhage: a helpful problem solving tool. Med Ultrason 2022;31;24(3):284–289. 10.11152/mu-3454 35437529

[2] Friedrich-Rust M, Schneider G, Bohle RM, Herrmann E, Sarrazin C, Zeuzem S, Bojunga J. Contrast-enhanced sonography of adrenal masses: differentiation of adenomas and nonadenomatous lesions. AJR Am J Roentgenol 2008;191(6):1852–60. 19020259 10.2214/AJR.07.3565

[3] Miele V, Piccolo CL, Galluzzo M, Ianniello S, Sessa B, Trinci M. Contrast-enhanced ultrasound (CEUS) in blunt abdominal trauma. Br J Radiol 2016;89(1061):20150823. 26607647 10.1259/bjr.20150823 PMC4985457

[4] Yusuf GT, Sellars ME, Deganello A, Cosgrove DO, Sidhu PS. Retrospective Analysis of the Safety and Cost Implications of Pediatric Contrast-Enhanced Ultrasound at a Single Center. AJR Am J Roentgenol 2017;208(2):446–452. 27959665 10.2214/AJR.16.16700

[5] Holmes JF, Lillis K, Monroe D, Borgialli D, Kerrey BT, Mahajan P, Adelgais K, Ellison AM, Yen K, Atabaki S, Menaker J, Bonsu B, Quayle KS, Garcia M, Rogers A, Blumberg S, Lee L, Tunik M, Kooistra J, Kwok M, Cook LJ, Dean JM, Sokolove PE, Wisner DH, Ehrlich P, Cooper A, Dayan PS, Wootton-Gorges S, Kuppermann N; Pediatric Emergency Care Applied Research Network (PECARN). Identifying children at very low risk of clinically important blunt abdominal injuries. Ann Emerg Med 2013;62(2):107–116.e2. 23375510 10.1016/j.annemergmed.2012.11.009

[6] Savoia P, Jayanthi SK, Chammas MC. Focused Assessment with Sonography for Trauma (FAST). J Med Ultrasound 2023;19;31(2):101–106. 10.4103/jmu.jmu_12_23 PMC10413405 37576415

[7] Holmes JF, Kelley KM, Wootton-Gorges SL, Utter GH, Abramson LP, Rose JS, Tancredi DJ, Kuppermann N. Effect of Abdominal Ultrasound on Clinical Care, Outcomes, and Resource Use Among Children With Blunt Torso Trauma: A Randomized Clinical Trial. JAMA 2017;13;317(22):2290–2296. 10.1001/jama.2017.6322 PMC5815005 28609532

[8] Riera A, Hayward H, Torres Silva C, Chen L. Reevaluation of FAST Sensitivity in Pediatric Blunt Abdominal Trauma Patients: Should We Redefine the Qualitative Threshold for Significant Hemoperitoneum? Pediatr Emerg Care 2021;1;37(12):e1012–e1019. 10.1097/PEC.0000000000001877 31356479

[9] Nti BK, Benzoni N, Starr R, Hays M, Vish D, End B, Russell F. Serial Trauma Abdominal Ultrasound in Children (STAUNCH): A Pilot Study. Pediatr Emerg Care 2024;1;40(9):623–626. 10.1097/PEC.0000000000003208 38587011

[10] Valentino M, De Luca C, Galloni SS, Branchini M, Modolon C, Pavlica P, Barozzi L. Contrast-enhanced US evaluation in patients with blunt abdominal trauma. J Ultrasound 2010;13(1):22–7. 23396012 10.1016/j.jus.2010.06.002 PMC3552645

[11] Miglioretti DL, Johnson E, Williams A, Greenlee RT, Weinmann S, Solberg LI, Feigelson HS, Roblin D, Flynn MJ, Vanneman N, Smith-Bindman R. The use of computed tomography in pediatrics and the associated radiation exposure and estimated cancer risk. JAMA Pediatr 2013;1;167(8):700–7. 23754213 10.1001/jamapediatrics.2013.311 PMC3936795

[12] Sidhu PS, Sellars ME, Deganello A, Eds. Contrast-enhanced ultrasound in pediatric imaging. Berlin: Springer, 2021.

[13] Zakaria OM, Daoud MYI, Zakaria HM, Al Naim A, Al Bshr FA, Al Arfaj H, Al Abdulqader AA, Al Mulhim KN, Buhalim MA, Al Moslem AR, Bubshait MS, AlAlwan QM, Eid AF, AlAlwan MQ, Albuali WH, Hassan AA, Kamal AH, Majzoub RA, AlAlwan AQ, Saleh OA. Management of pediatric blunt abdominal trauma with split liver or spleen injuries: a retrospective study. Pediatr Surg Int 2023;9;39(1):106. 10.1007/s00383-023-05379-0 36757505

[14] Donner V, Thaler J, Hautz WE, Sauter TC, Ott D, Klingberg K, Exadaktylos AK, Lehmann B. Contrast-enhanced point of care ultrasound for the evaluation of stable blunt abdominal trauma by the emergency physician: A prospective diagnostic study. J Am Coll Emerg Physicians Open 2024;19;5(2):e13123. 10.1002/emp2.13123 PMC11031391 38644807

